# Hyperfertility, obesity, and stillbirth: new considerations for clinical practice

**Published:** 2009-05-30

**Authors:** Louis G. Keith, Tawanda Ngorima, Olha M. Tsar

**Affiliations:** 1 Department of Obstetrics and Gynecology, Feinberg School of Medicine, Northwestern University; Chicago, Illinois USA; 2 Department of Obstetrics & Gynecology, School of Medicine, Lviv National Medical University; Lviv, Ukraine; 3 The Sims Institute/Sims International Fertility Clinic; Dublin, Ireland

## Abstract

This research focuses on two well known phenomenon that regularly confront obstetricians on a worldwide basis. The first is *hyperfertility,* whose effects are well known within and outside the obstetrics community. The second is *obesity*, a problem of growing importance throughout the developed and developing world. Each is discussed in view of recently published evidence. In this work, we show how these two concepts interlock and how they represent a substantial clinical challenge to all physicians providing care to reproductive aged women.

## Introduction

### Hyperfertility

A prime example of the maternal effects of hyperfertility is the Empress Mumtaz for whom the Taj Mahal was built as a memorial following her death from post partum hemorrhage after her fourteenth obstetric delivery. The public is aware of this memorial in the form of a UNESCO world heritage monument; the obstetric community is aware of the tragedy that preceded its construction. Despite this, the effects of hyperfertility on fetal outcomes are neither well known nor well studied.

With few exceptions, definitions of parity differ in the obstetric literature. These include the term *nullipara,* a gravida with no prior pregnancy history. A *primipara* is a woman who has given birth to one child. However, confusion arises with the term *multipara,* because this term simply but ambiguously means a woman who has given birth to more than one child. Since many women have had numerous live births, ranging from two to >20, the literature often uses modifications of the primary term, including *grand multipara* and *great grand multipara.* Herein lies the confusion, because no standardization of this taxonomy exists at present. Thus, the parity reclassification system proposed in the thesis dissertation of Dr. Muktar Aliyu (Univ. Alabama-Birmingham USA) has real value [see Table 1], in that it permits more exact comparisons across discrete, clinically relevant groups for assessment of maternal and fetal outcomes [1–3].

The literature definition of “high” (>5) parity, based on ten studies from nine nations published between 1954 and 2001, presents interesting as well as unexpected findings. Thirty percent of these deliveries occurred in the United Arab Emirates, 11% in Nigeria, 5% in Trinidad, and 0.6% in Croatia and Hong Kong, respectively. A search for the term “great grand multipara (>10 prior live births) revealed 11 studies published between 1992 and 2002 from 6 nations, 7 of which were from the Middle East. Of interest, definitions varied considerably, as did study sizes (range: 139–2709); both factors contributed to ascertainment biases. In addition, non adjustment for confounding variables frequently led to methodological biases.

The literature associating multiparity with adverse maternal outcomes is enormous. For example, between 1865 and 2004 at least 37 studies emanating from 17 nations variously mentioned one or more of the following adverse maternal outcomes as being related to multiparity: uterine rupture, chronic renal disease, hypertensive disease, placenta previa, preeclampsia, uterine inertia/atony, anemia, post partum hemorrhage, abruptio placenta and, finally, diabetes. Though this list is long, it is by no means exclusive. Moreover, it is regularly affected by three factors which, if not controlled for, confound the relationship between high parity and adverse maternal outcomes. These are: 1) selection bias, i.e., low social economic status; 2) maternal age; and 3) the now widely appreciated fact that as one ages, diseases or adverse conditions tend to accumulate. In contrast, when fetal outcomes and multiparity were studied in 38 publications from 13 nations between 1940 and 2004, only 4 adverse fetal outcomes were mentioned repeatedly: preterm delivery, low birthweight, perinatal mortality, and stillbirth.

### Calculating BMI

**Figure 1:**
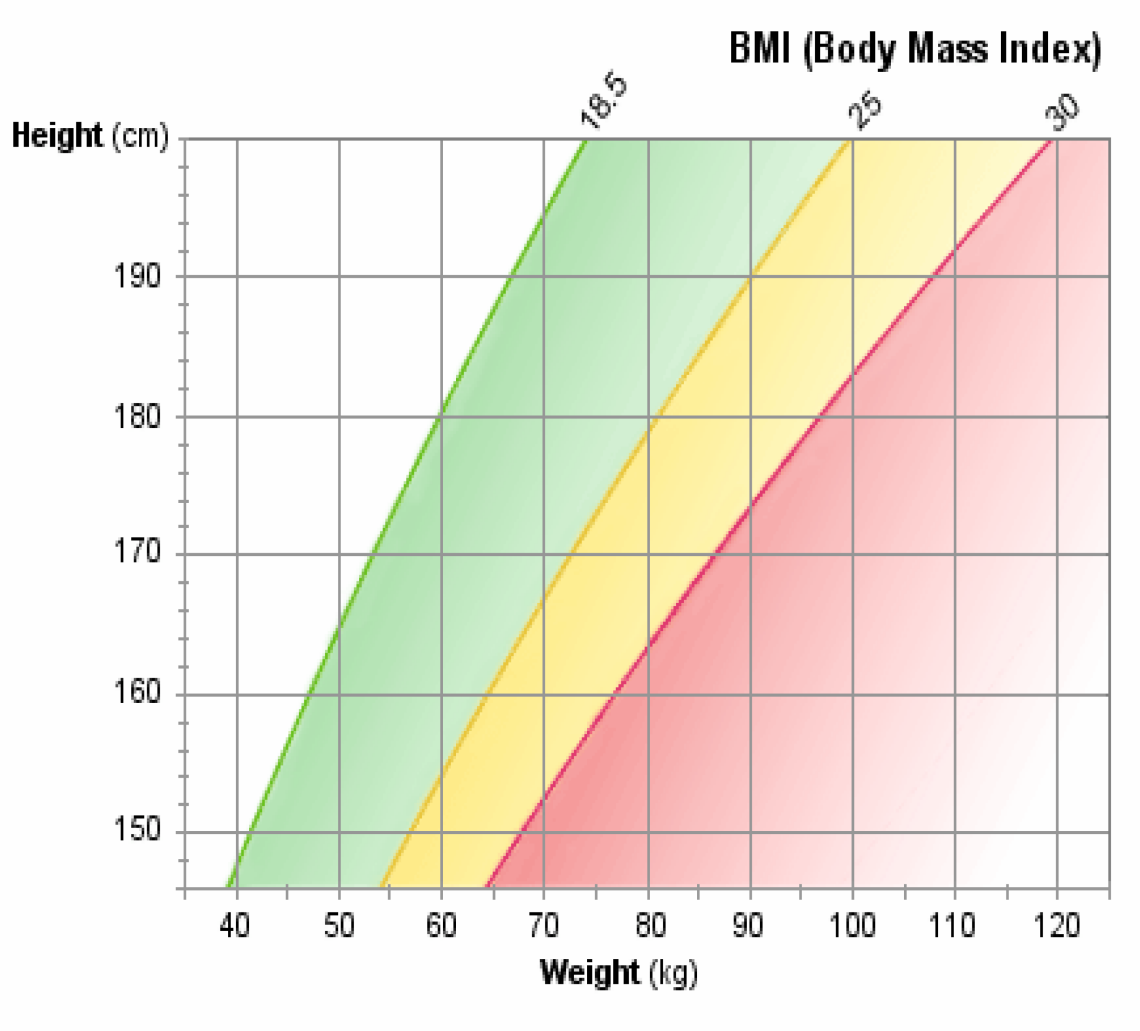
Body mass index calculation and classification.

Central to any discussion of obesity is determining body mass index (BMI). The importance of this clinical determination is recognized worldwide, not only for research but as a means of following patients who are trying to gain or to lose weight. Even when weight is not a consideration for change, BMI is a valuable construct that allows the patient to be placed into a discrete, clinically relevant group for study and assessment of progress and outcomes. BMI is an internationally accepted standard for weight comparisons that considers both height and weight. It can be described in SI units [BMI = weight in kg ÷ height^2^ (m^2^)], U.S. units [BMI = 703 × weight (lbs) ÷ height^2^ (inches^2^)], or U.K. units [BMI=6.35x weight (stone) ÷ height^2^ (m^2^)]. BMI classification by WHO (2000) standard definitions is shown in Table 2, and Figure 1 depicts a weight classification chart as used in many clinics and patient education classes.

## Methodology

Combined obstetrical data files and records on fetal death from 1989 to 2000, inclusive, were obtained from the U.S. National Center for Health Statistics. A database was constructed using singleton live births and fetal deaths ≥20 weeks gestation. Gestational age was calculated from the last menstrual period and the date of birth was obtained from data entered in vital records. Stillbirths (SB/IUFD) at ≥ 20 weeks were classified into the following four categories: 1) term SB: ≥ 37 completed gestational weeks, 2) preterm SB: < 37 completed gestational weeks, 3) SGA SB: <10%ile of birthweight for gestational age; and 4) preterm SGA stillbirth. The entire sample included 42,318,674 births distributed as follows: (1989–1992) 11,897,787 births, (1993–1996) 15,199,699 births, and (1997–2000) 15,221,188 births.

## Results

**Figure 2:**
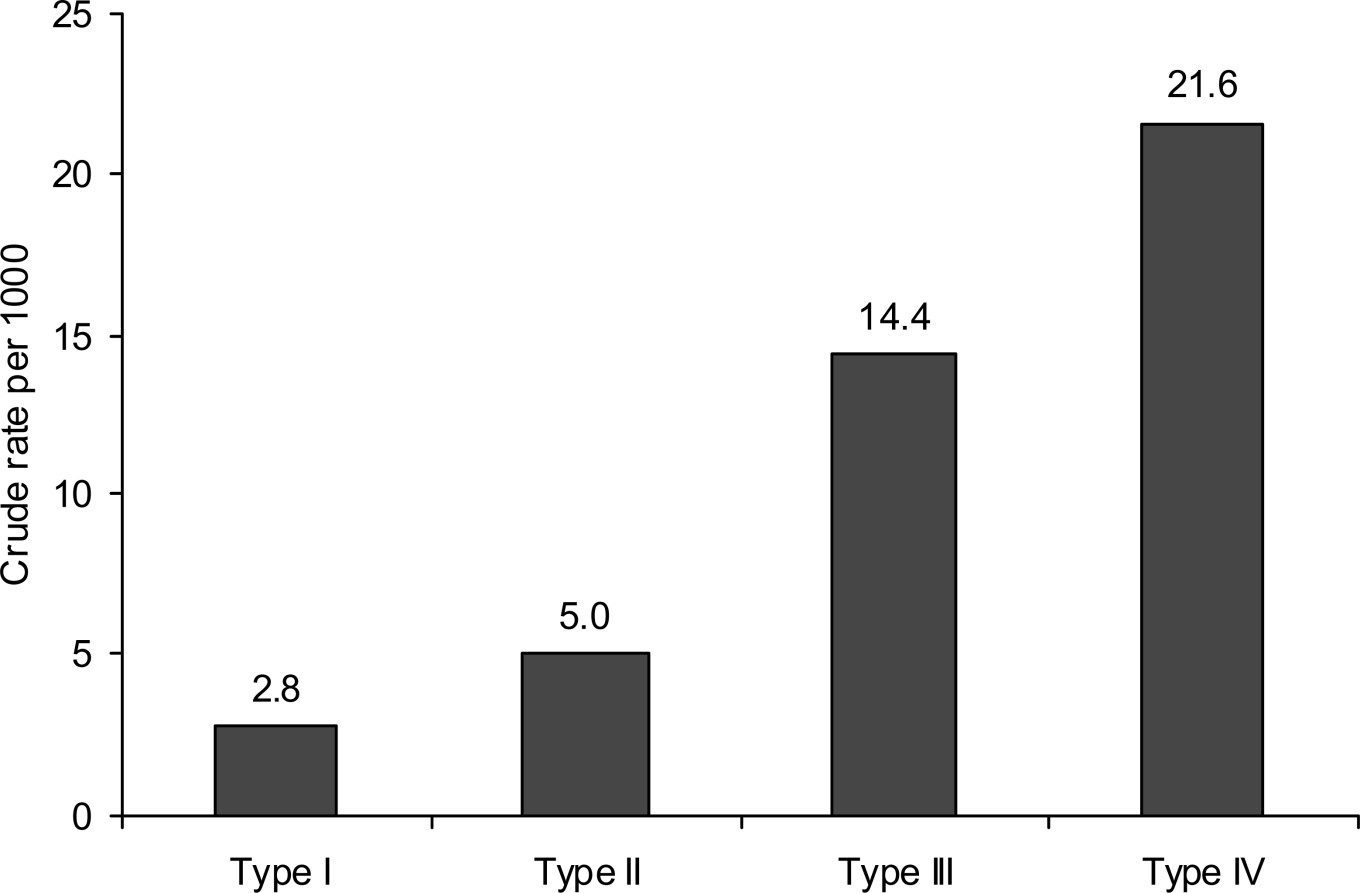
Crude stillbirth rate by fertility status (1989–2000).

**Figure 3:**
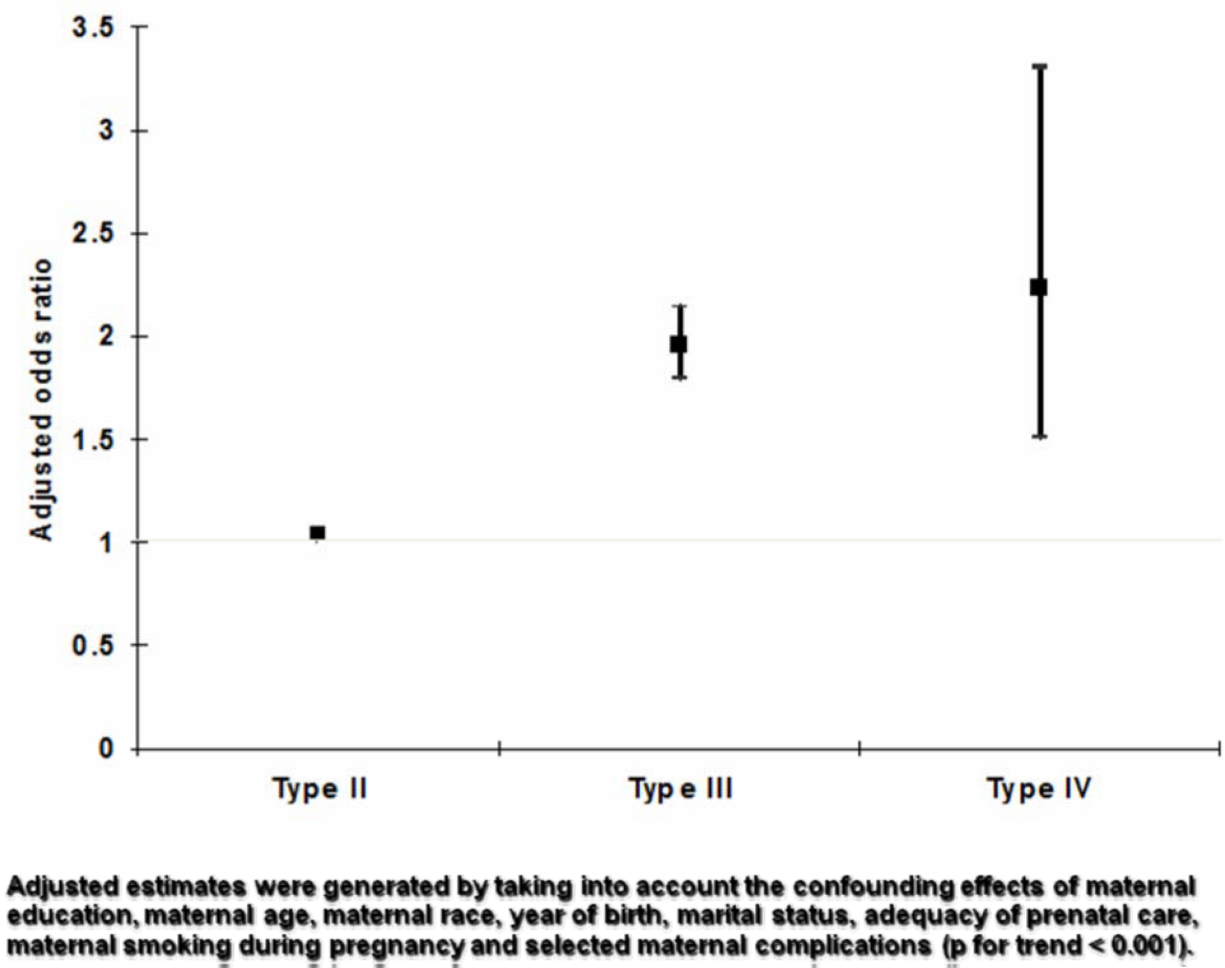
Adjusted odds ratios for stillbirth by fertility status (1989–2000).

**Figure 4:**
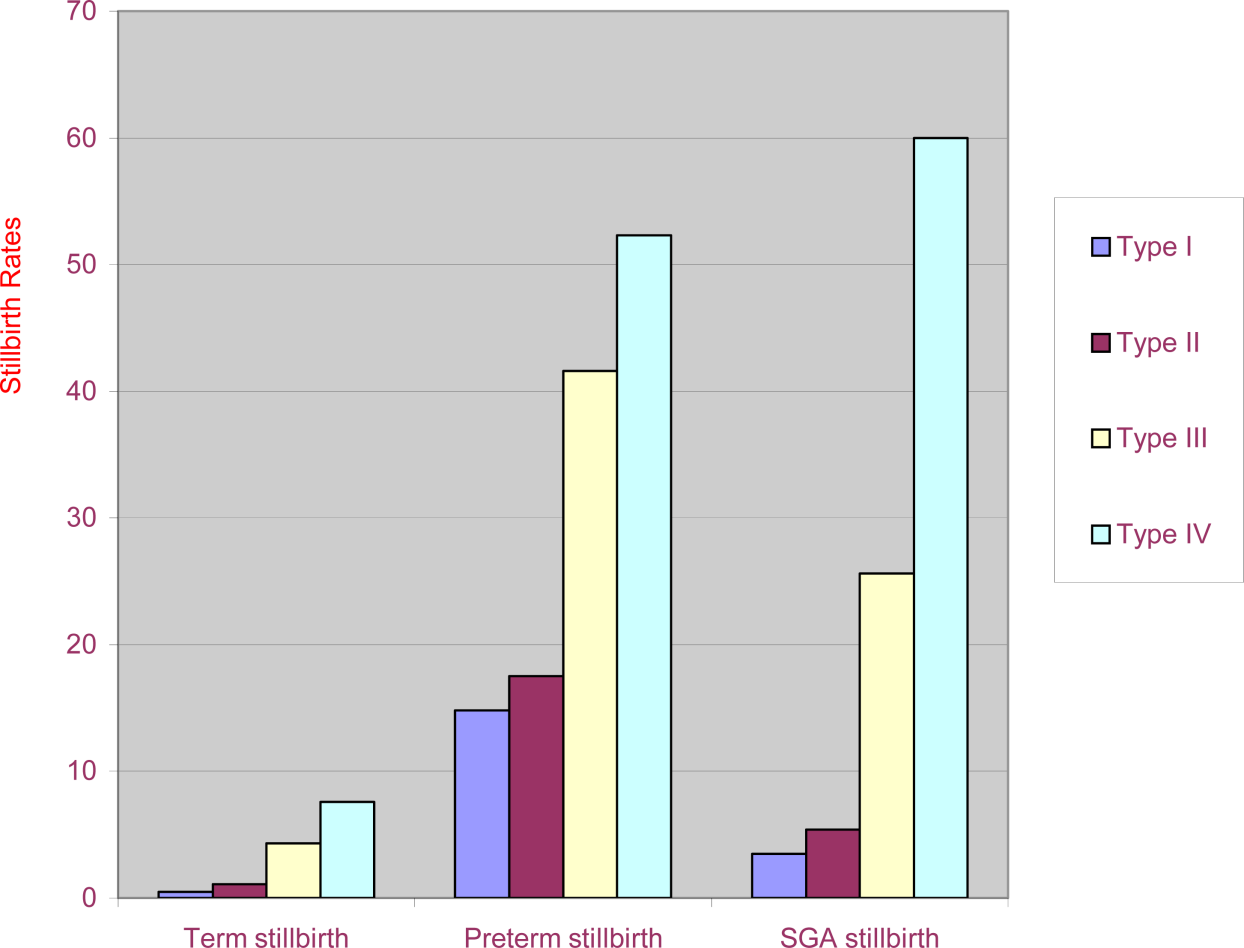
Type-specific stillbirth rate by fertility status (1989–2000)

**Figure 5:**
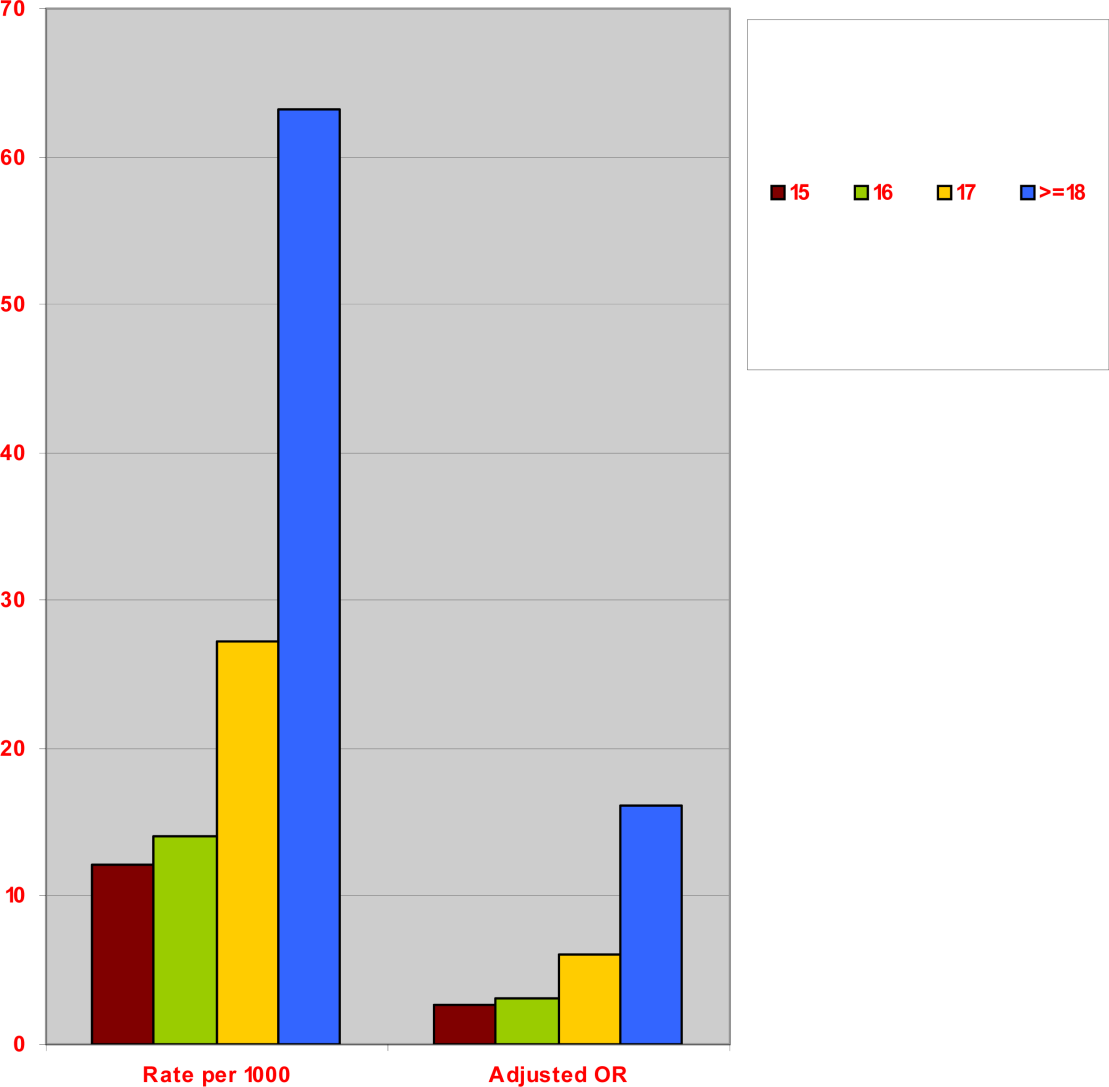
Stillbirth rates in Type IV obesity with dose effect (*p*<0.001, for trend).

Figure 2 presents the crude stillbirth rate per 1000 deliveries by fertility status, and Figure 3 shows the adjusted odds ratio by fertility status in the same study intervals. These estimates considered the confounding effects of maternal education, age and race, year of birth, marital status, and adequacy of prenatal care, maternal smoking during pregnancy, and selected maternal complications (*p*<0.001, for trend). The type-specific stillbirth rates (as defined above) for the same years are shown in Figure 4, while Figure 5 presents stillbirth rates in type 4 hyperfertility with a dose effect shown (*p*< 0.001, for trend).

## Discussion

No simple explanation fits the findings of this study, although several come to mind and are distinct possibilities. Foremost among these is micronutrient depletion, a condition that has received inadequate study in pregnant women, particularly as a function of parity status. “Maternal Depletion Syndrome” is a term often used to describe the physiologic status of the grand multipara in countries where undernourishment is common. Whether this would apply directly to some patients in the United States is not clear. However, it is unrealistic to think that a woman would not lose significant amounts of vital minerals and micronutrients if repeated pregnancies occur without sufficient time for restoration of maternal health. While it is generally regarded that a minimum of 18 months are required for health restitution after pregnancy, it was not possible to ascertain intra-partum intervals in our data set.

If knowledge of maternal depletion is poor, an understanding of local effects of repeated pregnancies on the uterine milieu is even more deficient. Although difficult to prove, it is plausible that uterine “overexhaustion” could lead to fetal under-nutrition via presence of scar tissue at prior placental sites. Maternal age and disease state may also affect fetal outcomes, but neither has been studied specifically in the hyperfertile population, and the prospects of doing so are slim given the large numbers of diseases that would need assessment and the small number of women of hyperfertile women who deliver on an annual basis.

This analysis derives from data obtained from very large populations, which minimized selection biases and provided acceptable levels of precision in the estimates. These data help clinicians understand the link between extreme fertility and the risk of fetal demise, thereby enhancing their ability to counsel all patients who may be considering large families. At the same time, study limitations must be acknowledged, including our lack of access to autopsy data or definitive cause of death for the patients included here. This deficiency could have complicated interpretation of results from investigations reviewing smaller populations, but we doubt that this represented a major negative impact on the present study. Second, no data were available regarding birth spacing or domestic activities/lifestyle which may have been associated with preterm labor. The same may be said for the absence of information on negative birth behaviors or psychosocial stressors, which may have been present in those mothers who experienced stillbirths. Additionally, no data were available to designate patient religion or other personal belief system. If it could be presumed that hyperfertility is more often associated with certain religious systems, the diverse and multi-religious composition of the American population might provide at least five such groups, if not more, that contribute to the cohort of women having 15 or more children. These might include certain Catholics, Orthodox Jews, Mormons, Hutterites, and fundamentalist Christians. Under such circumstances, it would be irresponsible to suggest that hyperfertility in the United States is based upon a given religious theology.

Stillbirth risk increases incrementally with ascending fertility in hyperfertile women, implying a dose effect relationship. Women who are moderately fertile (2–4) have the lowest risk of stillbirth; women who are hyperfertile (≥15) have the highest risk. These facts should be imparted to all multiparous women as part of the prenatal counseling process.

## Obesity

Unlike other areas of medicine where statistics are kept by governments and global organizations, no accurate data exist to describe the number of the world’s inhabitants who are overweight, obese, or morbidly obese. Obesity is present to such an extent in many developed countries and in some urban areas of developing nations that it is now considered by some health authorities as more dangerous to the public health than smoking.

This problem is closely related to overeating, although this approach belies the complex interplay among numerous social and economic factors that impact lifestyle. For example, present eating habits have evolved in response to the worldwide increase in gross national product since the 1950’s, the increasing reliance on pre-prepared meals (especially in the developed nations), and the popularity of fast foods and sweetened drinks which all too often depend on high-fructose corn syrup for their sweet taste and their unseen caloric burden.

Undoubtedly the obesity epidemic adversely affects males as well as females. In the latter group, however, some of its worst complications occur in those who are severely obese and simultaneously pregnant. It is now estimated that 22% of women of reproductive age and 54% of women aged 20–39 are either overweight or obese. The morbid effects of obesity on mothers has gained considerable attention recently, and has resulted in the publication of the first ever monograph on the topic [4]. In contrast, the adverse effect of extreme obesity on fetal outcomes has received far less attention.

## Methodology

This study utilized the public health records maintained by the State of Missouri. We reviewed maternally-linked cohort data files from 1978–1997 (inclusive), which provided delivery data linked to biological mothers and sibships. All recorded singleton livebirths and fetal deaths ≥ 20 weeks were evaluated. Stillbirth was defined as an intrauterine death at ≥ 20 weeks’ gestation. Multiple pregnancies were excluded; race/ethnicity was confined to non- Hispanic blacks and non-Hispanic whites. Maternal age was categorized as <35 or ≥ 35. A two-tailed test was used to assess the null hypothesis, with type I error set at 5%; logistic regression was used as required. Exclusions included multiple gestations (38,981–2.4%), pregnancies < 20 weeks and >44 weeks (76,305–4.8%), no BMI data (29.092–1.8%). A total of 1,413,953 mother-offspring pairs met inclusion criteria and were available for analysis: BMI ≤ 30 (134,527 – 9.5%); Class 1 Obesity (83,254 – 5.9%); Class 2 Obesity (33.364 – 2.3%); and Class 3 Obesity (17,909 – 1.3%). This methodology was used to test three basic hypotheses: 1) high maternal BMI leads to *in utero* fetal demise; 2) the relationship between maternal BMI and fetal demise is dose dependent; and 3) among obese gravidas, black/white disparity in fetal death widened with increasing BMI.

## Results

Table 3 illustrates the overwhelming preponderance of risk of complications in obese compared to non obese gravidas. Whereas Table 3 is based on USA data, Table 4 is derived from Israeli data. Here, the Odds Ratios are significantly elevated for other adverse obstetric outcomes in obese compared to non obese individuals. The USA data summarized in Table 5 shows risk of stillbirth by obesity subtype, along with crude and adjusted hazard ratios with 95% confidence intervals. These data show that extreme obesity doubles the risk of stillbirth compared to the rate found among normal-weight women.

Because of traditions established prior to WWII in reporting racial differences in U.S. government statistics, such categories remain in modern times. Table 6 presents crude and adjusted hazard ratios (95% CI) for stillbirths among black and white populations, respectively. Starting with overall obesity and ending with extreme obesity, black gravidas have a significantly higher stillbirth risk compared to whites (*p*<0.01, for trend).

## Discussion

These findings provide new data concerning risk of obesity in terms of adverse fetal outcomes. On the one hand, this is not surprising considering the ever-increasing understanding of the health consequences of obesity. Yet, they are also instructive from a medical education perspective because the likelihood that any one medical provider in London or Chicago (the media-designated “obesity capitals of the world”) will see enough stillbirths in individual practice to make the same conclusions is exceedingly small, if not impossible. Thus, our information offers real and substantive proof that governmental and societal forces must urgently act to reduce the health burden of obesity.

This investigation has two advantages. First, the fact that it is population-based and from a state jurisdiction whose data is regularly used by U.S. health authorities for assessing the nation’s health significantly minimizes the possibility of ascertainment bias. In addition, the large sample size provides an acceptable level of precision in the estimates. Despite these advantages, however, certain limitations must be acknowledged. For example, we used vital statistics obtained over a twenty-year assessment period, although data gathered over such a long study interval could itself represent a type of ascertainment bias. Also, the infant cohorts over this twenty year period were exposed to different obstetric practices as the specialty of obstetrics evolved. Data from these different infant cohorts were aggregated and analyzed as if they were entirely uniform, and this might be regarded as a methodological bias. It should be noted, however, that two recent publications from Scandinavia (both of which are cited by Oron *et al.* in *Obesity and Pregnancy* [4]) assessed a Danish birth cohort of 54,505 women and documented a five fold increase in the stillbirth rate among obese women. A Swedish population-based cohort (167,750 women) documented an OR for late fetal death of 3.2 [1.2–6.2] for overweight women and 4.3 [2.0 −9.3] for obese women. These reports are consistent with the findings observed in the present study.

## Conclusion

Stillbirth risk increases incrementally with ascending fertility in hyperfertile women, implying a dose effect relationship. Women who are moderately fertile (2–4) have the lowest risk of stillbirth; women who are hyperfertile (≥15) have the highest risk. These facts should be imparted to all multiparous women as part of the prenatal counseling process. The findings of our work can be reasonably extrapolated to other populations and indeed are supported by data from Sweden and Denmark. These findings provide healthcare members of the healthcare team with strong evidence to bring before governmental and societal bodies proposing various means for reducing the incidence of obesity within society.

## Figures and Tables

**Table 1:** Reclassification of parity according to the University of Alabama-Birmingham (USA) model.

**Reclassification of Parity: the UAB Model**		
Previous live births	Fertility Class	Definition
2–4	I	Moderately fertile
5–9	II	Very fertile
10–14	III	Extremely fertile
≥ 15	IV	HYPERFERTILE

**Table 2:** Obesity designations by body mass index (BMI).

CLASSIFICATION	BMI
Normal	18.5–24.9
Overweight	25–29.9
Obese class 1	30.00–34.99
Obese class 2	35.00–39.99
Obese class 3	>40.00

**Table 3:** Risk of obstetrical complication by obesity vs. non-obesity.

	Obese (80,599) %	Non-obese (548,040) %	*P*
Insulin-dependent diabetes	1.6	0.4	<0.01
Other forms of diabetes	4.7	1.5	<0.01
Chronic hypertension	3.0	0.5	<0.01
Pre-eclampsia	8.4	3.4	<0.01
Eclampsia	0.2	0.1	<0.01

**Table 4:** Reproductive and perinatal features as a function of maternal weight.

Characteristics	Obese (n=1769)	Non-obese (n=124311)	OR	95%CI	P
Meconium stained amniotic fluid	21.5%	16.2%	1.4	1.2–1.6	<.001
Recurrent abortions	7.1%	4.8%	1.5	1.2–1.9	<.001
Birth weight >4000 grams	6.4%	4.5%	1.5	1.2–1.8	<.001
Non-vertex presentation	9.2%	5.9%	1.6	1.3–1.9	<.001
Cesarean delivery	27.8%	10.8%	3.2	2.9–3.5	<.001

**Table 5:** Stillbirth risk, by obesity subtype.

	Stillbirths (n)	*Crude hazard ratio (95% CI)	*Adjusted hazard ratio (95% CI)
Normal weight (BMI =18.5 – 24.9)	7,091	1.0	1.0
Obesity (all subtypes)	1149	1.5 (1.4–1.6)	1.4 (1.3–1.5)
Class I obesity (BMI =30 – 34.9)	649	1.4 (1.3–1.5)	1.3 (1.2–1.4)
Class II obesity (BMI =35 – 39.9)	290	1.5 (1.3–1.7)	1.4 (1.3–1.6)
Extreme Obesity	210	2.0 (1.8–2.4)	1.9 (1.6–2.1)

**Table 6:** Stillbirth rate in obese women, by race.

	Black			White		
	Stillbirths (n)	CHR (95% CI)	AHR (95% CI)	Stillbirth (n)	CHR (95% CI)	AHR (95% CI)
Overall obesity	320	2.4 (2.1–2.7)	1.9 (1.7–2.1)	829	1.6 (1.5–1.8)	1.4 (1.3–1.5)
Class I obesity	168	1.9 (1.6–2.2)	1.6 (1.4–1.9)	481	1.2 (1.1–1.3)	1.3 (1.2–1.4)
Class II obesity	81	2.1 (1.7–2.6)	1.9 (1.5–2.3)	209	1.3 (1.2–1.5)	1.4 (1.2–1.6)
Extreme obesity	71	2.7 (2.1–3.4)	2.3 (1.8–2.9)	139	1.8 (1.5–2.1)	1.8 (1.6–2.2)
*p* (for trend)	<0.01					
